# Prognostic value of inflammatory markers NLR, PLR, and LMR in gastric cancer patients treated with immune checkpoint inhibitors: a meta-analysis and systematic review

**DOI:** 10.3389/fimmu.2024.1408700

**Published:** 2024-07-10

**Authors:** Shufa Tan, Qin Zheng, Wei Zhang, Mi Zhou, Chunyan Xia, Wenzhe Feng

**Affiliations:** ^1^ Shaanxi University of Traditional Chinese Medicine the First Clinical Medical College, Shaanxi, China; ^2^ Fuling District Zhenxi Central Health Center, Inpatient Department, Chongqing, China; ^3^ Physical Examination Center of Fuling Hospital Affiliated to Chongqing University, Chongqing, China; ^4^ Anorectal Department, Affiliated Hospital of Shaanxi University of Traditional Chinese Medicine, Shaanxi, China

**Keywords:** gastric cancer, immune checkpointin hibitors, inflammatory markers, survival, meta-analysis

## Abstract

**Background:**

Immune checkpoint inhibitors (ICIs) represent a groundbreaking approach to cancer therapy. Inflammatory markers such as the neutrophil-to-lymphocyte ratio (NLR), platelet-to-lymphocyte ratio (PLR), and lymphocyte-to-monocyte ratio (LMR) have emerged as potential indicators strongly associated with tumor prognosis, albeit their prognostic significance remains contentious. The predictive value of NLR, PLR, LMR in patients with gastric cancer (GC) treated with ICIs has not been fully explored; therefore, we conducted a meta-analysis to examine the potential of inflammatory markers NLR, PLR, and LMR as survival predictors in this population.

**Methods:**

A comprehensive search was conducted across PubMed, Embase, Web of Science, and Cochrane databases, with the search cut-off date set as March 2024. Hazard ratios (HR) and their corresponding 95% confidence intervals (CI) were calculated to assess the prognostic significance of NLR, PLR, and LMR for both progression-free survival (PFS) and overall survival (OS).

**Results:**

Fifteen cohort studies involving 1336 gastric cancer patients were finally included in this meta-analysis. The results of the meta-analysis showed that high levels of NLR were associated with poorer OS and PFS in GC patients receiving ICIs, with combined HRs of OS [HR=2.01, 95%CI (1.72,2.34), P<0.01], and PFS PFS[HR=1.59, 95%CI (1.37,1.86), P<0.01], respectively; high levels of PLR were associated with poorer OS and PFS, and the combined HR was OS [HR=1.57, 95%CI (1.25,1.96), P<0.01], PFS [HR=1.52,95%CI (1.20, 1.94), P<0.01], respectively; and there was an association between elevated LMR and prolonged OS and PFS, and the combined HR was OS [HR=0.62, 95%CI (0.47,0.81), P<0.01], and PFS [HR=0.69, 95%CI (0.50,0.95), P<0.01].

**Conclusion:**

In gastric cancer (GC) patients treated with immune checkpoint inhibitors (ICIs), elevated neutrophil-to-lymphocyte ratio (NLR) and platelet-to-lymphocyte ratio (PLR) were associated with poorer overall survival (OS) and progression-free survival (PFS), while high lymphocyte-to-monocyte ratio (LMR) was linked to improved OS and PFS. Subgroup analyses suggested that NLR might be particularly pertinent to the prognosis of GC patients. In conclusion, the inflammatory markers NLR, PLR, and LMR serve as effective biomarkers for prognostic assessment in GC patients, offering valuable insights for therapeutic decision-making in the realm of GC immunotherapy. Prospective studies of high quality are eagerly awaited to validate these findings in the future.

**Systematic review registration:**

https://www.crd.york.ac.uk/PROSPERO/#myprospero, identifier CRD42024524321.

## Introduction

Gastric cancer (GC) presents a significant global health challenge, ranking as the fourth most common cancer worldwide after lung, colorectal, and liver cancers (1). According to GLOBOCAN 2020, around 1.1 million new cases of gastric cancer were diagnosed in 2020, representing 5.6% of all cancer cases, with approximately 800,000 deaths, accounting for 7.7% of all cancer-related deaths (2). However, due to its subtle onset and rapid progression, most patients with gastric cancer receive a diagnosis of advanced-stage tumors at the time of initial diagnosis. Platinum-based dual chemotherapy regimens such as XELOX (oxaliplatin+capecitabine), FOLFOX (oxaliplatin+calcium folinic acid+fluorouracil), SOX (oxaliplatin+tiglialasole), and FP (fluorouracil+cisplatin) are preferred as first-line therapeutic options. Despite some improvement in overall survival rates with these regimens, the median survival remains limited to 8-10 months, with a persistently low 5-year survival rate (3, 4). Systemic treatments for gastric cancer include chemotherapy, immunotherapy, and targeted therapy, with the combination of immune checkpoint inhibitors and chemotherapy emerging as a standard treatment for advanced gastric cancer patients (5). Immune checkpoints (ICPs) are integral components of the immunosuppressive network, playing a vital role in modulating the intensity and specificity of the immune response to prevent excessive activation. In normal circumstances, immune cells like T cells, dendritic cells (DCs), macrophages, and natural killer cells (NK) identify and eliminate tumor cells while protecting normal cells from harm (6). However, during tumor progression, ICPs are often upregulated, triggering various immune checkpoint pathways on immune cells. This activation results in the suppression of immune cell function, hindering the body’s anti-tumor immune response and aiding tumor evasion from immune surveillance (7). Immune checkpoint inhibitors (ICIs) work by enhancing the patient’s innate immune response through the blockade of co-inhibitory signaling pathways mediated by immune checkpoints such as programmed death-1 (PD-1)/programmed death ligand 1 (PD-L1), cytotoxic T-lymphocyte-associated antigen 4 (CTLA-4), lymphocyte activation gene-3 (LAG-3), and others, which are critical for regulating immune tolerance (8). By inhibiting these pathways, ICIs restore the functionality of immune cells against tumors, thereby enhancing the immune system’s ability to exclude and eliminate tumor cells (9). Several studies have demonstrated that ICI shows good anticancer activity in a variety of cancers, including melanoma (10), non-small cell lung cancer (11), renal cell carcinoma (12), esophageal cancer (13) and hepatocellular carcinoma (14). Nivolumab and Pembrolizumab, two FDA-approved IgG4 monoclonal antibodies that target the programmed death-1 (PD-1) receptor, have emerged as the mainstay treatment for individuals with unresectable or advanced GC.

The advent of Immune Checkpoint Inhibitor (ICI) therapies has revolutionized the approach to treating malignant tumors. However, not all patients derive equal benefits from immunotherapy, with some experiencing severe side effects and substantial treatment costs (15). Therefore, there is an urgent need to identify specific patient populations that are most likely to respond positively to ICIs. This emphasizes the crucial role of biomarker identification in gastric cancer patients who may exhibit a favorable response to ICI therapy (16). Currently, biomarkers such as PD-L1 positivity, microsatellite instability (MSI)/mismatch repair (MMR), EBV, and tumor mutational load (TMB) are used in gastric cancer patients receiving immunotherapy to predict the effectiveness of immunosuppressive drugs (17). However, these biomarkers are insufficient to meet current clinical needs, highlighting the need for more accurate predictive markers. Studies have indicated that specific inflammatory markers in peripheral blood can reflect the interaction between host inflammation, immunity, and tumors. Furthermore, pro-inflammatory molecules produced by the systemic inflammatory response through innate immune cells can promote tumor growth and spread while activating oncogenic signaling pathways (18). Multiple studies have affirmed (19, 20) the ability of peripheral blood markers, specifically the neutrophil-to-lymphocyte ratio, monocyte-to-lymphocyte ratio, and platelet-to-lymphocyte ratio, to reflect the overall inflammatory status of the body. These markers are non-invasive, cost-effective, easily accessible, and convenient. They have been employed to gauge the immune and inflammatory condition in patients with various malignant tumors, such as non-small-cell lung cancer (21) and malignant melanoma (22), which is important for the clinical diagnosis and prognostic evaluation of cancer. To date, there has been no comprehensive meta-analysis conducted on the inflammatory markers NLR, PLR, and LMR. Therefore, we conducted this meta-analysis to evaluate the prognostic significance of inflammatory markers in gastric cancer patients treated with immune checkpoint inhibitors.

## Materials and methods

**Figure 1:**
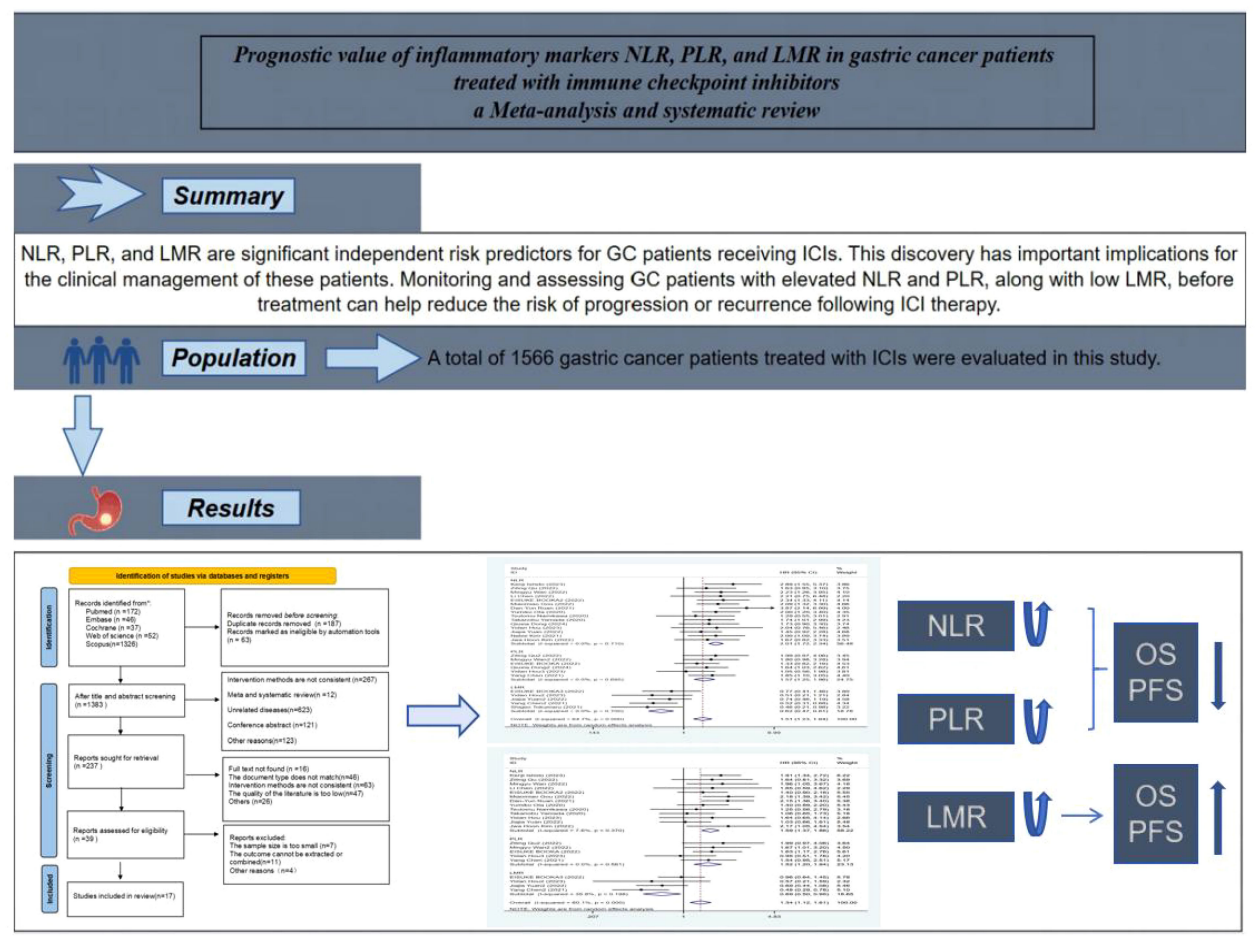
Graphical abstract of the meta-analysis.

The protocol has been registered in the International Prospective Register of Systematic Reviews data base (PROSPERO: CRD42024524321). The article draw Graphical abstract in this paper, as shown in **Figure 1**.

### Literature search strategy

Two researchers (TSF, ZQ) independently searched using Pubmed, Scopus, Embase, Web Of Science, and Cochrane databases.Mesh terms in PubMed were used to broaden the search, with search terms including “Neutrophil-lymphocyteratio”, “Platelet-lymphocyteratio”, “lymphocyte-monocyteratio”, “ Stomach Neoplasm”, “Gastric Neoplasms”, “Cancerof Stomach”, “Stomach Cancers”, “Gastric Cancer”, “Gastric Tumor”, “Immune Check point Inhibitor”, “Immune Check point Blockers”, “PD-L1Inhibitors”. “Programmed Death-Ligand1Inhibitors”, “Pembrolizumab”, “Tremelimumab”, “Nivolumab” had no restriction on language or study type in the search strategy, and the last date of retrieval was 1 March 2024. The titles and abstracts of the retrieved articles were reviewed by two authors, and the articles were screened according to the Inclusion and exclusion criteria. Data on basic information about the relevant literature, study objectives, results and follow-up were extracted by one of the authors and reviewed by the second author, and in case of disagreement, they were judged by a third-party expert. Systematic evaluation was performed according to the Preferred Reporting Items for Systematic Evaluation and Meta-Analysis (PRISMA) guidelines (23).

### Inclusion and exclusion criteria

The inclusion criteria were as follows:

(1) Patients with clinically confirmed gastric cancer and treated with ICIs;
(2) Studies reporting the effect of high versus low expression of inflammatory markers NLR, PLR, and LMR on patient survival using risk ratios (HR) and 95% confidence intervals (CI);
(3) Literature in English and Chinese;
(4) The outcome metrics were overall survival (OS) or progression-free survival (PFS);
(5) Inclusion of study design as randomised controlled trials, observational studies, cross-sectional studies, retrospective studies or prospective studies;

The exclusion criteria are as follows:

(1) Failure to provide survival information;
(2) Lack of adequate data or results;
(3) Duplicate publications or incomplete information;
(4) Non-comparative studies, animal experiments, reviews, letters, guidelines, case reports, pathomechanisms, conference abstracts, expert opinions, editorials, and commentaries;
(5) Literature in other languages;

### Data extraction

Two researchers independently screened the literature based on predefined inclusion and exclusion criteria. Information was then extracted using a standardized data extraction form and cross-checked by the two researchers individually. Disagreements were resolved through discussion. Studies lacking relevant data were excluded. For each study, the following information was gathered: (1) study characteristics including first author, country, year of publication, study type, duration, immune checkpoint inhibitors utilized, and critical values; (2) patient baseline information including the number of patients, their age, and gender; and (3) study outcomes, specifically hazard ratio (HR) values for overall survival (OS) and progression-free survival (PFS).

### Literature quality assessment

The quality of the included cohort studies was independently assessed using the Newcastle-OttawaScale (NOS, 24), which consists of three metrics: cohort selection, comparability, and outcome assessment. The modified NOS is a 9-star scale with 1-3 stars for low quality studies, 4-6 stars for moderate quality, and 7-9 stars for high quality. Scoring was done independently by two investigators, and third-party experts were consulted to resolve any large differences between their scores or if this affected the study’s inclusion in the final analysis.

### Statistical analysis

StataSE15.0 software was used for statistical analysis and combined HR and 95% confidence interval (95% CI) were calculated, P<0.05 showed significant difference between the two groups. Heterogeneity was evaluated using I² values, I²≤30%, 30% < I²< 75% and ≥75% were considered to indicate low, medium and high heterogeneity respectively (25). I²< 50% was analyzed using a fixed-effects model, while I²≥50% was analyzed using a random-effects model (26, 27). Sensitivity analyses were performed for outcomes with high heterogeneity and the source of heterogeneity was analyzed, and the presence of publication bias was assessed using Begg’s funnel plot and Egger’s test (28, 29), with P > 0.05 indicating the absence of publication bias.

## Results

### Literature search results

**Figure 2:**
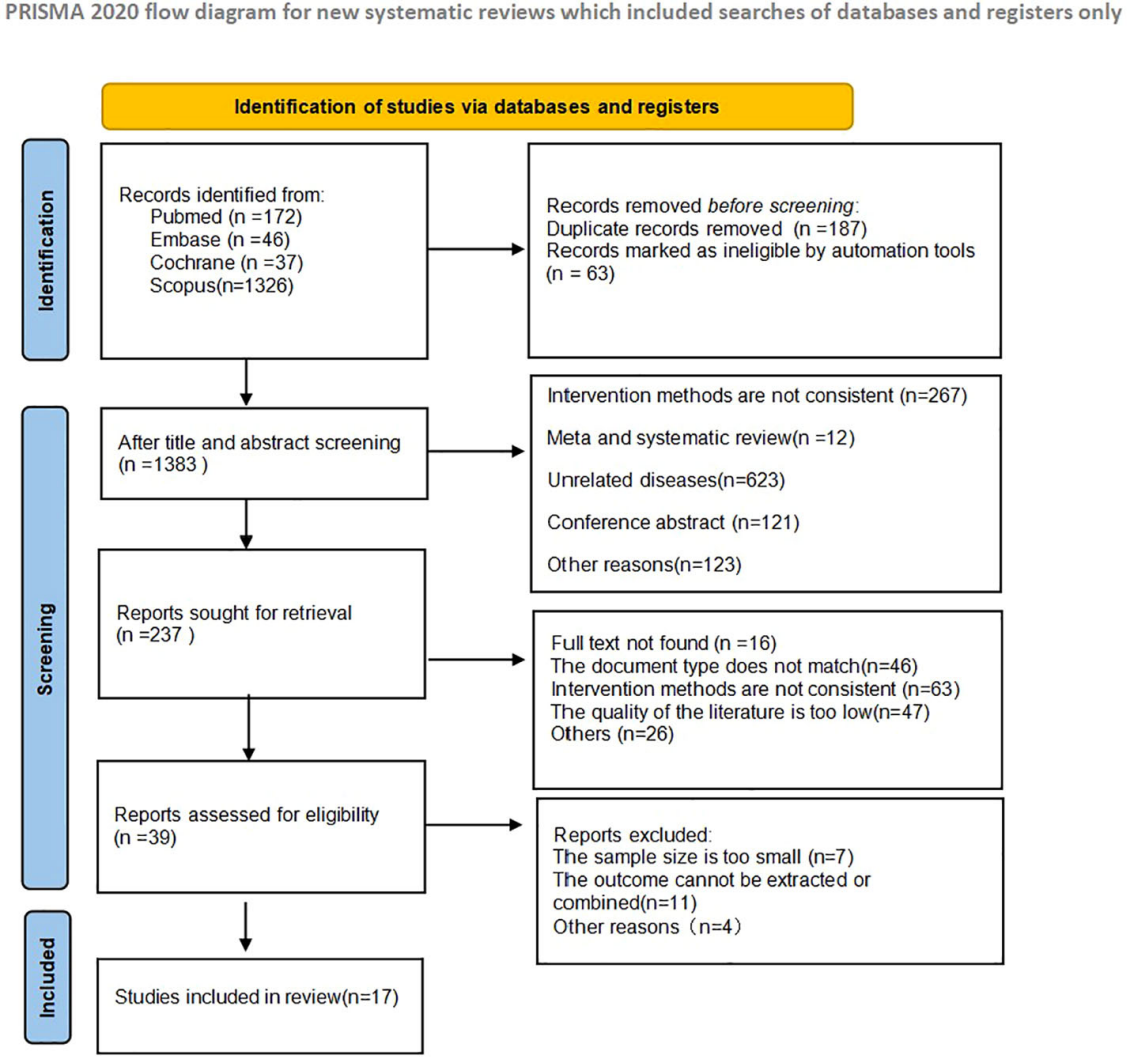
Schematic diagram of literature search criteria and including studies in meta-analyses.

A total of 1633 articles were retrieved in the initial literature search. 187 duplicate studies were excluded; after reading the article titles and abstracts, 1396 were excluded based on the nerf criteria and 237 studies were initially included. Subsequently, we read the full text and excluded 220 studies that did not meet the inclusion criteria. 17 studies were finally included in the Meta-analysis, and the literature screening process and results are shown in **Figure 2**.

### Basic characteristics of the included studies

As shown in **Table 1**, a total of 1566 gastric cancer patients treated with ICIs were evaluated in the 17 included studies, and all 17 studies were cohort studies, of which 16 were retrospective cohort studies and 1 was a prospective cohort study. There were multiple inflammatory markers studied in one study in the included literature, so we numbered the same literature different inflammatory markers. The study characteristics, patient baseline, and study results of the included studies are displayed in **Table 1**.

**Table 1:** Clinical and demographic characteristics of studies included in the meta-analysis.

Firstauthor	Year	Researchtype	Authorstates	Sample size	Age	Sex(Male/female)	Duration	ICIsagents	Follow-up (months)	Cut-off	Markers	Survival outcome
KenjiIshido (30)	2023	Cohort(retrospective)	China	59	71 (43–86)	45\14	2017-2020	Nivolumab	24	1.5	NLR	PFS, OS
ZitingQu (31)	2022	Cohort(retrospective)	China	106	≤65: 77 (72.6)<65: 29 (27.4)	72\34	2019-2021	anti-PD-1	24	3.11	NLR	PFS, OS
ZitingQu2	2022	Cohort(retrospective)	China	106	≤65: 77 (72.6)<65: 29 (27.4)	72\34	2019-2021	anti-PD-1	24	243.33	PLR	PFS, OS
MingyuWan (32)	2022	Cohort(retrospective)	China	45	≤60: 34 (76)<60: 11 (23)	35\10	2017-2020	Anti-PD-1+ chemo	36	3.85	NLR	PFS, OS
MingyuWan2	2022	Cohort(retrospective)	China	45	≤60: 34 (76)<60: 11 (23)	35\10	2017-2020	Anti-PD-1+ chemo	36	214.08	PLR	PFS, OS
LiChen (33)	2022	Cohort(retrospective)	China	106	61.0 (53.3,66.0)	74\32	2016-2020	anti-PD-1	60	3	NLR	PFS, OS
EISUKEBOOKA (34)	2022	Cohort(retrospective)	Japan	61	71 (46-86)	49\12	2017-2021	Nivolumab Pembrolizumab	12	3.9	NLR	PFS, OS
EISUKEBOOKA2	2022	Cohort(retrospective)	Japan	61	71 (46-86)	49\12	2017-2021	Nivolumab Pembrolizumab	12	118	PLR	PFS, OS
EISUKEBOOKA3	2022	Cohort(retrospective)	Japan	61	71 (46-86)	49\12	2017-2021	Nivolumab Pembrolizumab	12	2.3	LMR	PFS, OS
MiaomiaoGou (35)	2022	Cohort(retrospective)	China	137	NA	98\39	2016-2020	Nivolumab Pembrolizumab Toripalimab Sintilimab	12	3.23	NLR	PFS, OS
Dan-YunRuan (36)	2021	Cohort(prospective)	China	58	60 (52–66)	41\17	2016-2017	Toripalimab	15	2.7	NLR	PFS, OS
YumikoOta (37)	2020	Cohort(retrospective)	Japan	98	66 (33–84)	68\30	2014-2018	Nivolumab	15	3	NLR	PFS, OS
TsutomuNamikawa (38)	2020	Cohort(retrospective)	Japan	29	71 (49–86)	19\10	2017-2019	Nivolumab	25	2.5	NLR	PFS, OS
TakanobuYamada (39)	2020	Cohort(retrospective)	Japan	89	NA	42\47	2017-2019	Nivolumab	12	2.5	NLR	PFS, OS
QiuxiaDong (40)	2024	Cohort(retrospective)	China	197	NA	160\37	2020-2022	Nivolumab Toripalimab Sintilimab	26	NA	NLR	OS
QiuxiaDong2	2024	Cohort(retrospective)	China	197	NA	160\37	2020-2022	Nivolumab Toripalimab Sintilimab	26	NA	PLR	OS
YidanHou (41)	2023	Cohort(retrospective)	China	77	≤60: 41<60: 36	53\24	2020-2022	Sintilimab Camrelizumab Tislelizumab	24	2.3	NLR	PFS, OS
YidanHou2	2023	Cohort(retrospective)	China	77	≤60: 41<60: 36	53\24	2020-2022	Sintilimab Camrelizumab Tislelizumab	24	3.4	LMR	PFS, OS
YidanHou3	2023	Cohort(retrospective)	China	77	≤60: 41<60: 36	53\24	2020-2022	Sintilimab Camrelizumab Tislelizumab	24	149	PLR	PFS, OS
JiajiaYuan (42)	2022	Cohort(retrospective)	China	80	60 (54–66)	61\19	2014-2019	Anti-PD-(L)1	30	NA	NLR	PFS, OS
JiajiaYuan2	2022	Cohort(retrospective)	China	80	60 (54–66)	61\19	2014-2019	Anti-PD-(L)1	30	NA	LMR	PFS, OS
YangChen (43)	2021	Cohort(retrospective)	China	139	60 (51–67)	103\36	2015-2019	Anti-PD-(L)1+ chemo/anti-VEGF/anti- HER	24	173.7	PLR	PFS, OS
YangChen2	2021	Cohort(retrospective)	China	139	60 (51–67)	103\36	2015-2019	Anti-PD-(L)1+ chemo/anti-VEGF/anti- HER	24	3.5	LMR	PFS, OS
ShigeoTokumaru (44)	2021	Cohort(retrospective)	Japan	55	69 (40–84)	39\16	2017-2020	Nivolumab	12	3.28	LMR	OS
Nalee Kim (45)	2021	Cohort(retrospective)	South Korea	185	59 (47–70)	120\65	2016-2019	Nivolumab Pembrolizumab+ chemo	12	3	NLR	OS
Jwa Hoon Kim (46)	2022	Cohort(Prospective)	South Korea	45	60 (23–76)	34\11	2014–2016	Nivolumab	12	2.9	NLR	OS, PFS

(NLR, Neutrophil-lymphocyteratio; PLR, Platelet-lymphocyteratio; LMR, lymphocyte-monocyteratio; OS, overallsurvival; PFS, progression-freesurvival; anti-PD-1, ogrammeddeath-(ligands)1; NA, Not mentioned in the original article).

### The quality assessment of the included studies

The quality of the included cohort studies was evaluated using the Newcastle-Ottawa Scale (NOS) for quality and the results are shown in **Table 2**.

**Table 2:** NOS quality evaluation table.

Study	Selection	Comparability	Outcomes	Total
	1 2 3 4		123	
Kenji Ishido	★★★	★	★★★	7
ZitingQu	★★★	★	★★	6
MingyuWan	★★★	★	★★★	7
LiChen	★★★	★	★★	6
EISUKEBOOKA	★★★	★★	★★★	8
MiaomiaoGou	★★★	★★	★★★	8
Dan-YunRuan	★★★	★	★★★	7
Yumiko Ota	★★★★	★	★★★	8
Tsutomu Namikawa	★★★	★★	★★★	8
Takanobu Yamada	★★★★	★	★★★	8
QiuxiaDong	★★★	★★	★★★	8
YidanHou	★★★	★	★★★	7
JiajiaYuan	★★	★	★★★	6
YangChen	★★★	★★	★★★	8
ShigeoTokumaru	★★★	★	★★★	7
Nalee Kim	★★★	★★	★★★	8
Jwa Hoon Kim	★★★★	★★	★★★	9

★ represents the score, and one ★ is one point.

### Meta-analysis results

#### Overall survival

**Figure 3:**
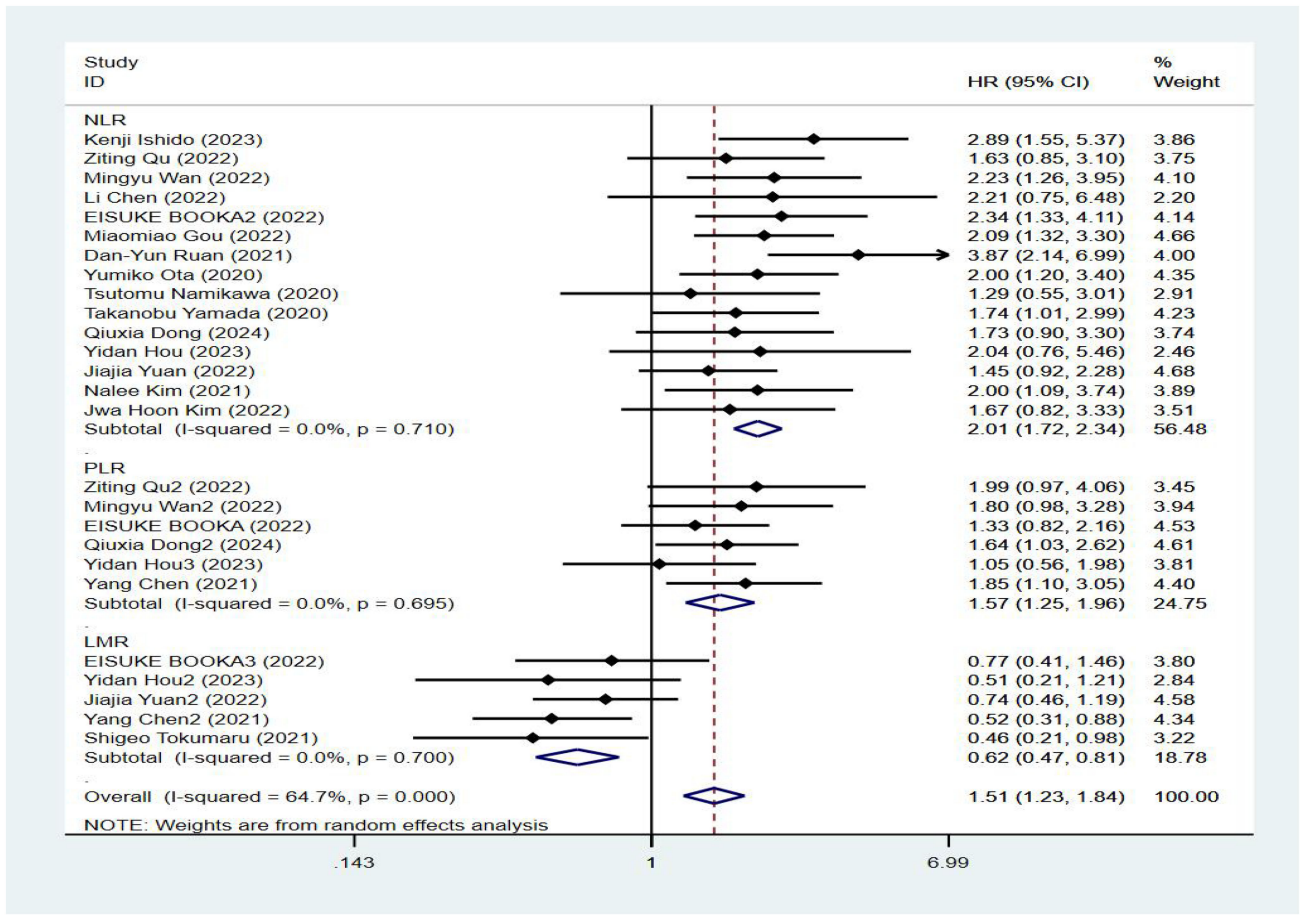
Forest plot of overall survival (OS) data.

Seventeen studies reported patient overall survival, and **Figure 2** shows the forest plots of hazard ratios determined in the 17 studies, taking into account the large heterogeneity between studies (P < 0.01, I²=64.7%). Therefore, we conducted a meta-analysis using a random-effects model. We divided different inflammatory factors into subgroups for analysis, and the results showed that regardless of inflammatory markers, they were effective in predicting the survival of patients with gastric cancer, and the difference was statistically significant. Among them, high NLR was associated with poor OS, and the combined HR was OS[HR=2.01, 95% CI(1.72,2.34), P < 0.01]. High PLR was associated with poor OS, and the combined HR was specific OS[HR=1.57, 95% CI(1.25,1.96), P < 0.01]. Subgroup analysis showed that high NLR may be associated with worse survival prognosis of GC patients. There was an association between elevated LMR and prolonged OS, and the combined HR was OS[HR=0.62, 95% CI(0.47,0.81), P < 0.01], respectively, as shown in **Figure 3**.

#### Progression-free survival

**Figure 4:**
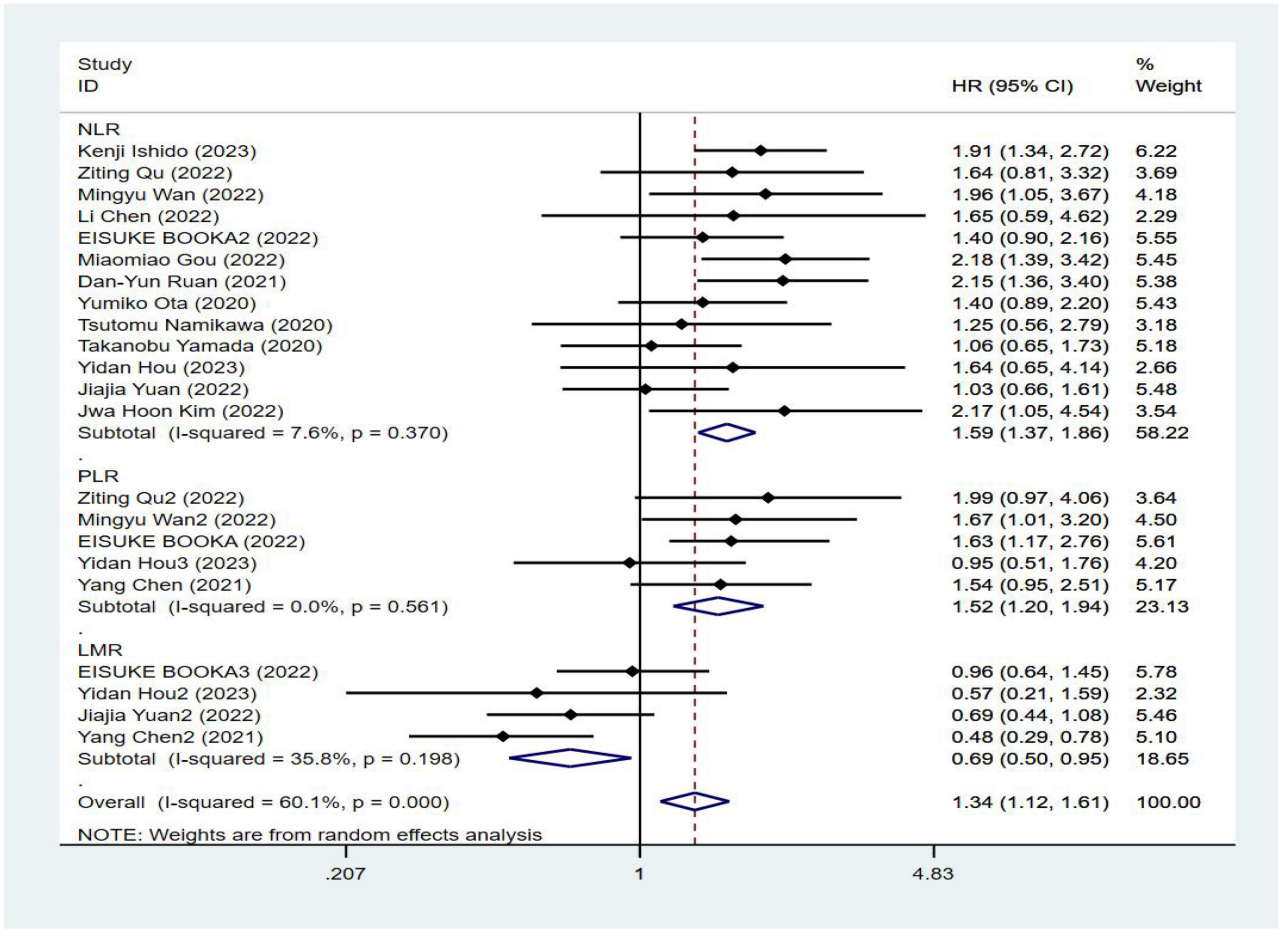
Forest plot of progression-free survival (PFS) data.

Fourteen studies reported progression-free survival of patients, and **Figure 3** shows a forest plot of the risk ratios identified in the 14 studies, taking into account the large heterogeneity between studies (P < 0.01, I²= 60.1%). Therefore, we performed a meta-analysis using a random effects model. We divided different inflammatory factors into subgroups for analysis, which showed that whatever inflammatory markers were effective in predicting PFS in gastric cancer patients, with statistically significant differences, in which high levels of NLR were associated with worse PFS, with a combined HR of PFS, respectively [HR=1.59, 95%CI (1.37,1.86), P<0.01]; high levels of PLR were associated with poorer PFS, the combined HR was PFS [HR=1.52, 95%CI (1.20,1.94), P<0.01], and subgroup analysis showed that high NLR might be associated with poorer survival prognosis in patients with GC. There was an association between higher LMR and prolonged PFS, the combined HR was PFS [HR=0.69, 95%CI (0.50,0.95), P<0.01], and the subgroup analysis showed that high NLR might be associated with poorer survival prognosis in patients with GC, see **Figure 4**.

### Sensitive analysis

**Figure 5:**
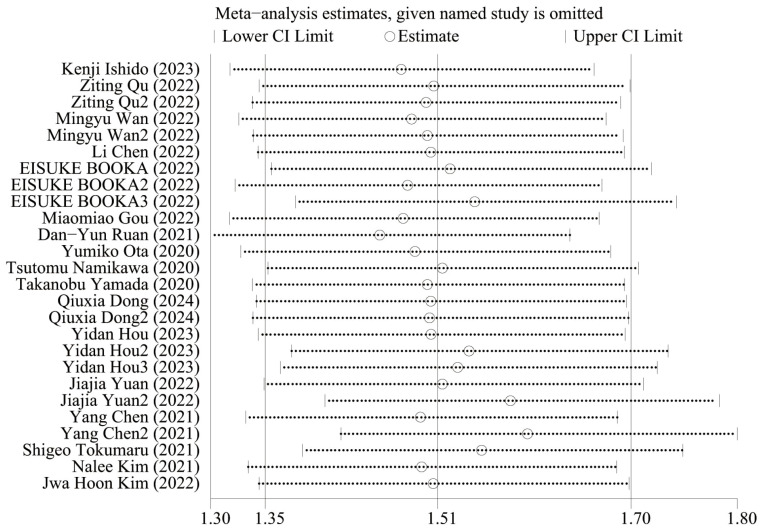
Sensitivity analysis of OS.

**Figure 6:**
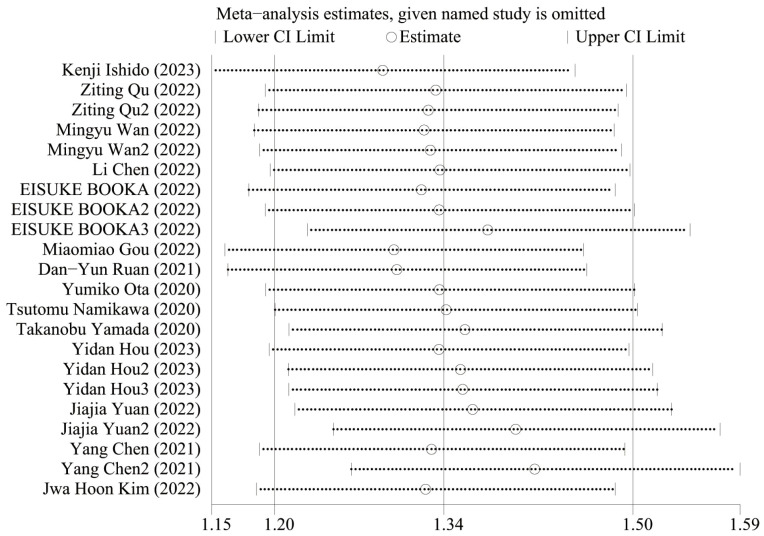
Sensitivity analysis of PFS.

**Figure 5** shows the OS sensitivity analysis, where the effect sizes remained consistent within the original range after removing each study in turn, indicating a robust and reliable model. **Figure 6** shows the PFS sensitivity analysis, with low sensitivity, indicating a robust and reliable model.

### Publication bias

**Figure 7:**
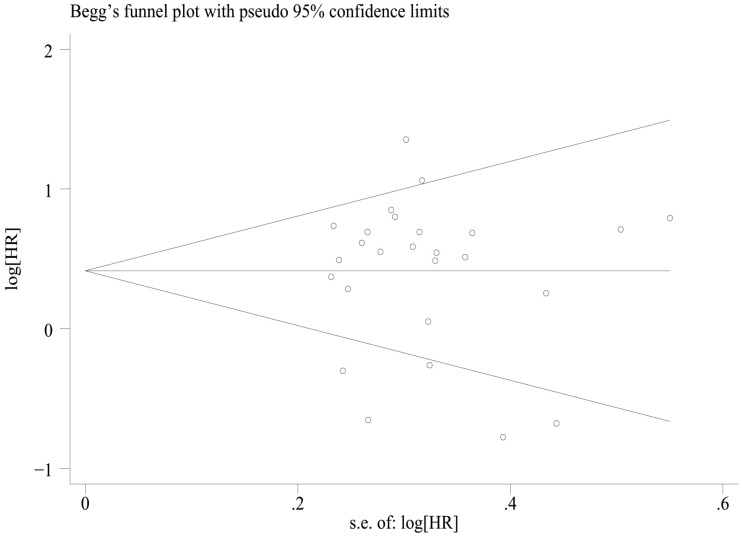
Funnel plot for the evaluation of publication bias for OS.

**Figure 8:**
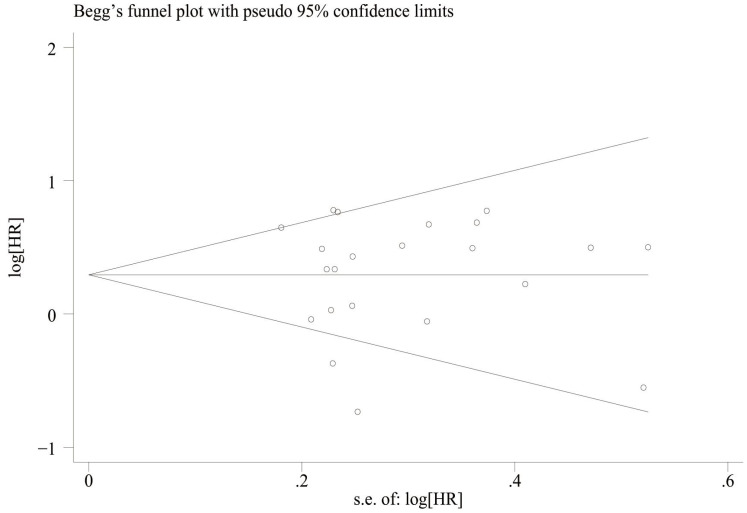
Funnel plot for the evaluation of publication bias for PFS.

We assessed publication bias by plotting funnel plots for OS and PFS, and the results showed that overall survival (**Figure 7**) Egger’sP=0.832, Begg’sP=0.597 indicated no significant publication bias; there was no significant asymmetry in the shape of the funnel plots, and all the studies fell within the 95% CI. PFS (**Figure 8**) Egger’sP=0.995, Begg’sP=0.910 indicating no significant publication bias; no significant asymmetry in funnel plot shape, all studies within 95% CI.

### Subgroup analysis

To determine the source of the heterogeneity in OS and PFS, we performed separate subgroup analyses for NLR, PLR, and LMR. Our results showed that elevated NLR was an important prognostic factor for poor OS and PFS, independent of country, sample size, cutoff value, study type, follow-up time and combination of drugs (P < 0.05; **Table 3**). Elevated LMR was consistently associated with improved OS and PFS in various parameters, including country, sample size, follow-up time, combination therapy, and cutoff value (p < 0.05; **Table 4**). PLR rise is also bad OS and predictors of PFS, and the country, the sample size, the critical value, the follow-up time and combination therapy (P < 0.05; **Table 5**).

**Table 3:** The HR for OS and PFS of NLR was pooled in subgroup analyses.

Subgroup	PFS				OS			
	Study	HR [95%CI]	P value	I^2^	Study	HR [95%CI]	P value	I^2^
Country								
China	8	1.75 (1.46,2.11)	P<0.001	7.2	9	2.08 (1.71,2.55)	P<0.001	7.8
Japan	4	1.28 (1.00,1.65)	0.049	0	4	1.90 (1.41,2.54)	P<0.001	0
South Korea	1	2.17 (1.04,4.51)	0.038	NA	2	1.84 (1.16,2.93)	0.009	0
Sample size								
<100	10	1.53 (1.30,1.79)	P<0.001	17.2	10	2.04 (1.69,2.47)	P<0.001	0
≥100	3	1.96 (1.37,2.8)	P<0.001	0	5	1.91 (1.45,2.52)	P<0.001	0
cut-off								
≥3	6	1.66 (1.33,2.07)	P<0.001	0	6	2.06 (1.65,2.57)	P<0.001	0
<3	6	1.69 (1.37,2.10)	P<0.001	17	6	2.22 (1.69,2.92)	P<0.001	29.5
Study design								
Retrospective	11	1.51 (1.29,1.77)	P<0.001	2.9	11	1.90 (1.61,2.25)	P<0.001	0
Prospective	2	2.16 (1.46,3.17)	P<0.001	0	2	2.82 (1.84,4.32)	P<0.001	56.4
Follow-up								
≤15	6	1.62 (1.33,197)	P<0.001	33.1	5	2.25 (1.77,2.85)	P<0.001	19.7
>15	7	1.55 (1.25,1.93)	P<0.001	0	10	1.83 (1.49,2.25)	P<0.001	0
Combined medication								
Monotherapy	8	1.52 (1.28,1.81)	P<0.001	21.1	9	1.95 (1.59,2.39)	P<0.001	19.7
Combined therapy	5	1.76 (1.35,2.31)	P<0.001	0	6	2.08 (1.63,2.65)	P<0.001	0

NA, Not Applicable.

**Table 4:** The HR for OS and PFS of LMR was pooled in subgroup analyses.

Subgroup	PFS				OS			
	Study	HR [95%CI]	P value	I^2^	Study	HR [95%CI]	P value	I^2^
Country								
China	3	0.58 (0.42,0.80)	P<0.001	0	3	0.61 (0.44,0.84)	0.003	0
Japan	1	0.96 (0.63,1.44)	0.845	NA	2	0.62 (0.38,1.02)	0.06	2.3
Sample size								
<100	3	0.80 (0.60,1.07)	0.137	0	4	0.65 (0.47,0.90)	0.009	0
≥100	1	0.48 (0.29,0.78)	0.004	NA	1	0.52 (0.30,0.87)	0.014	NA
cut-off								
≥3	2	0.49 (0.31,0.77)	0.002	0	3	0.50 (0.34,0.73)	P<0.001	0
<3	1	0.96 (0.63,1.44)	0.845	NA	1	0.77 (0.41,1.45)	0.42	NA
Follow-up								
≤15	1	0.96 (0.63,1.44)	P<0.001	NA	2	0.62 (0.38,1.02)	0.06	2.3
>15	3	0.58 (0.42,0.80)	0.01	0	3	0.61(0.44,0.84)	0.003	0
Combined medication								
Monotherapy	1	0.69 (0.44,1.08)	0.105	NA	2	0.64(0.43,0.97)	0.036	5.7
Combined therapy	3	0.71 (0.52,0.96)	0.126	57.1	3	0.59(0.41,0.85)	0.005	0

NA, Not Applicable.

**Table 5:** The HR for OS and PFS of PLR was pooled in subgroup analyses.

Subgroup	PFS				OS			
	Study	HR [95%CI]	P value	I^2^	Study	HR [95%CI]	P value	I^2^
Country								
China	4	1.47 (1.10,1.93)	0.009	0	5	1.63 (1.27,2.11)	P<0.001	0
Japan	1	1.63 (1.06,2.50)	0.026	NA	1	1.33 (0.81,2.15)	0.248	NA
Sample size								
<100	3	1.66 (1.11,2.49)	0.017	14	3	1.77 (1.30,2.42)	0.06	0
≥100	2	1.44 (1.06,1.95)	0.012	0	3	1.36 (0.98,1.88)	P<0.001	0
cut-off								
≥200	2	1.78 (1.14,2.80)	0.011	0	2	1.87 (1.18,2.97)	0.008	0
<200	3	1.42 (1.07,1.89)	0.015	6.6	3	1.41 (1.04,1.92)	0.026	0
Follow-up								
≤15	1	1.63 (1.06,2.50)	0.026	NA	1	1.33 (0.81,2.15)	0.248	NA
>15	3	1.47 (1.10,1.97)	0.009	0	5	1.63 (1.27,2.11)	P<0.001	0
Combined medication								
Monotherapy	1	1.98 (0.97,4.05)	0.059	NA	1	1.73 (0.82,3.87)	0.059	NA
Combined therapy	3	1.47 (1.13,1.89)	0.003	0	5	1.52 (1.20,1.93)	P<0.001	0

NA, Not Applicable.

## Discussion

Risk factors for gastric cancer primarily include Helicobacter pylori infection, dietary habits, tobacco use, obesity, and radiation exposure (47). While efforts to treat Helicobacter pylori have led to a decrease in global gastric cancer rates, the aging population trend suggests that future occurrence and mortality rates may remain high. Gastric cancer has a poor prognosis compared to other cancers, with global 5-year survival rates typically ranging from 20% to 40% (48). Chemotherapy is the standard treatment for advanced gastric cancer, with commonly used drugs like fluorouracil (5-Fu)/capecitabine, paclitaxel (with either paclitaxel or doxorubicin), and platinum compounds. However, the effectiveness of chemotherapy alone is limited, with a median overall survival of only 8 months for intermediate and advanced stages of gastric cancer (49). Immunotherapy has significantly improved cancer treatment, especially for gastric cancers with medium to high tumor mutational burden (TMB) and specific subtypes like MSI-H tumors or EBV-related cancers, which are more responsive to immunotherapy (50). The rapid progress in immune checkpoint inhibition has broadened the range of established combination chemotherapeutic approaches for esophagogastric adenocarcinoma (EGA). PD-1 inhibitors, such as nivolumab and pembrolizumab, have been approved for both single-agent and combination therapy in advanced gastric cancer, in first- or third-line treatment settings in Europe, the United States, and Taiwan, based on strong phase II and III trials (51). PD-1 is a negative co-stimulatory immune molecule found on the surface of T cells, B cells, and myeloid cells. Its ligand, PD-L1, is expressed on antigen-presenting cells (APCs) and various tumor cells. Higher levels of PD-L1 can trigger interleukin 10 secretion, resulting in T lymphocyte apoptosis and reduced calmodulin expression. This process enables tumor cells to evade the immune system and promotes epithelial-mesenchymal transition (52). The interaction between PD-L1 and PD-1 activates an immunosuppressive signaling pathway, leading to phosphorylation of the T-cell receptor (TCR) pathway, reduction in downstream activation signals, decreased cytokine production, and T-cell activation. This helps prevent T-lymphocyte over-activation, which could cause autoimmune diseases and tumor immune evasion. Additionally, this interaction influences the intensity and duration of the normal immune response (8, 53). PD-1/PD-L1 inhibitors have the potential to boost the immune response against tumors by blocking PD-1/PD-L1 interactions (54). Key inhibitors like pembrolizumab, nivolumab, and avelumab are currently used in clinical practice or in trials. Findings from the CheckMate-649 study (55), the largest randomized phase III trial in gastric cancer by the European Society for Medical Oncology (ESMO), revealed that combining nivolumab with chemotherapy significantly prolonged overall survival in patients with a PD-L1 combined positive score (CPS) of ≥5, reducing the risk of death by 20% (HR 0.80, 95% CI 0.71-0.90, P = 0.0002). In a refractory scenario, nivolumab, an anti-PD-1 antibody, showed a median overall survival of 5.26 months in the nivolumab group compared to 4.14 months in the placebo group in a phase III trial in Asia involving 493 patients from Japan, South Korea, and Taiwan (HR 0.63, 95% CI 0.51-0.78; p < 0.001) (56). The results of a Meta-analysis by Song Li (57) showed more benefit in patients with high dMMR/MSI-H and pd - l1 than in patients with low pMMR/MSS and pd - l1, and the combined results demonstrated that ICI-based neoadjuvant therapy for locally progressive gastric cancer has good efficacy and safety.

Current clinical trial results suggest that only a minority of patients experience benefits from PD-1/PD-L1 inhibitor therapy. Moreover, there are variations in treatment efficacy among patients with similar molecular profiles. Therefore, it is crucial to identify additional biomarkers and analyze them collectively to pinpoint the subset of patients most likely to benefit from immunotherapy. This strategy aims to optimize drug regimens for enhanced effectiveness. Inflammation plays a key role in contributing biologically active molecules to the tumor microenvironment, which is a critical factor in promoting tumor recurrence (58). Recent research has consistently shown a correlation between inflammation and tumorigenesis. Increased expression of inflammation-associated factors supports tumor progression, with these factors, such as inflammatory factors, inflammatory cells, and chemokines, being present in the microenvironment of early-stage tumors (59). Macrophages and neutrophils phagocytize pathogens, while dendritic cells, which are vital antigen-presenting cells, activate CD4+Th cells and CD8+T cells in lymph nodes. This orchestrates the adaptive immune system to eliminate pathogens, support tissue cell proliferation and repair, and maintain tissue homeostasis (60). However, inflammation triggered by oncogenic events persists as the organism cannot eradicate it, leading to chronic inflammation. Inflammatory cells and cytokines play crucial roles in regulating the growth, migration, and differentiation of tumor cells, fibroblasts, and endothelial cells within the tumor microenvironment. Many inflammatory mediators possess pro-angiogenic properties, induce cellular mutations and DNA damage, trigger inflammatory cascade responses, and cause tissue atrophy. The inflammatory state compromises the immune response, facilitating tumor immune escape and ultimately driving tumor progression and invasion (61, 62). It has been confirmed (63, 64) that peripheral blood inflammatory markers such as NLR, PLR, and LMR have been shown to reflect the systemic inflammatory status and are potential indicators for aiding in the clinical diagnosis and prognostic assessment of gastric cancer. Ogata et al. (65) research by Ogata et al. revealed that a high NLR before or after treatment with nabumab was linked to significantly shorter OS in patients with unresectable or recurrent gastric cancer. Dogan et al. (66) found that elevated PLR was associated with lower OS rates in patients with metastatic gastric cancer. Multiple studies have indicated (67, 68) that a higher preoperative LMR is correlated with improved DFS or OS in gastric cancer patients undergoing surgical resection, with critical values typically ranging from 3.15 to 5.15. However, conflicting results exist, as reported by Aldemir et al (69), who found that a high PLR did not impact the prognosis of patients with *in situ* gastric cancer. To address these discrepancies, a meta-analysis was conducted to investigate the predictive value of peripheral blood inflammatory markers for survival in gastric cancer patients receiving ICIs. According to the results of our meta-analysis, a high level of NLR was associated with poor OS and PFS in GC patients receiving ICIs. Merging the HR respectively OS [HR = 2.01, 95% CI (1.72, 2.34), P < 0.01], PFS [HR = 1.59, 95% CI (1.37, 1.86), P < 0.01]; High PLR was associated with poor OS[HR=1.57, 95%CI(1.25,1.96), P < 0.01] and PFS[HR=1.52, 95%CI(1.20, 1.94), P < 0.01]. Elevated LMR was associated with prolonged OS [HR=0.62, 95% CI(0.47,0.81), P < 0.01], PFS [HR=0.69, 95% CI(0.50, 0.95), P < 0.01], and OS[HR=0.62, 95% CI(0.47, 0.81), P < 0.01]. This indicates that NLR, PLR, and LMR are all independent predictors of survival in gastric cancer patients receiving ICIs. Subgroup analysis revealed that high NLR might result in poorer OS and PFS compared to high PLR. We deemed our results reliable based on the publication bias test. NLR represents the ratio of neutrophils to lymphocytes, with neutrophils, also termed polymorphonuclear cells (PMNs), constituting the most abundant leukocyte population in the body’s circulation, accounting for approximately 50-70% of all leukocytes (68). Tumor-associated neutrophils (TANs) secrete cytokines, chemokines, and related enzymes. These substances enhance the migration, invasion, and EMT of gastric cancer cells. TANs achieve this by promoting the release of vascular endothelial growth factor (VEGF) to facilitate the formation of tumor vasculature (70). TANs activate the JAK2/STAT3 pathway in gastric cancer cells by secreting interleukin-17a (IL-17a) (71). Neutrophils release DNA-toxic substances that cause DNA double-strand breaks and increase genomic instability, which are crucial in cancer initiation (72). Exosomes derived from gastric cancer cells prolong the survival of TANs, induce the expression of inflammatory factors, trigger autophagy, activate TANs through the HMGB1/TLR4/NF-κB signaling pathway, and enhance gastric cancer cell migration (73). An elevated neutrophil count indicates the host’s inflammatory state, while a decreased lymphocyte count suggests immunosuppression and weakened anti-tumor effects. The PLR reflects the ratio of platelet count to lymphocyte count. Platelets, originating from megakaryocytes in the bone marrow, are among the first responders at injury sites. Tumor cells adhere to each other, forming clusters that provide protection against high-flow shear stress and immune attacks. This adhesion not only promotes tumor cell proliferation and stability but also enhances their invasiveness (74). Neutrophils and platelets release vascular endothelial growth factor (VEGF) and transforming growth factor beta (TGF-β) to facilitate tumor metastasis. Platelets also play a role in promoting EMT, tumor cell survival in circulation, extravasation, colonization of distant sites, and chemo-resistance, all contributing to tumor cell proliferation (75, 76). Therefore, PLR is closely linked to tumor invasion, metastasis, and prognosis. On the other hand, LMR represents the ratio of lymphocyte count to monocyte count. Lymphocytes, key components of the adaptive and innate immune system, actively participate in immune surveillance, impeding tumor cell proliferation, invasion, and metastasis through their cytotoxic effects (77). A decrease in lymphocyte count hinders the lymphocyte-mediated anti-tumor immune response. Tumor-associated macrophages, originating from monocytes, accumulate in tumor tissues and create an inflammatory microenvironment. This microenvironment supports angiogenesis, tumor growth, and metastasis by suppressing the immune response, promoting neoangiogenesis, and breaking down the extracellular matrix. These processes facilitate tumor progression and distant migration (78). Lower lymphocyte levels in the tumor microenvironment weaken the body’s ability to resist tumors, thus aiding in tumor proliferation. On the other hand, higher LMR levels indicate a stronger immune system and improved tumor surveillance (79).

Our meta-analysis demonstrates several notable strengths: (1) It stands out as the most comprehensive study to date, integrating an extensive literature search, a larger sample size, and subgroup analysis for nuanced discussions; (2) The discussion section delves deeper into mechanistic aspects, setting it apart from prior meta-analyses; (3) We have validated the prognostic significance of NLR, PLR, and LMR in GC patients undergoing treatment with ICIs. Nonetheless, our study is not without limitations: (1) Variations in threshold definitions across studies may introduce bias; (2) The inclusion of studies exclusively from China and Japan warrants caution in extrapolating findings to other countries and regions.

## Conclusion

Our meta-analysis suggests that NLR, PLR, and LMR are significant independent risk predictors for GC patients receiving ICIs. This discovery has important implications for the clinical management of these patients. Monitoring and assessing GC patients with elevated NLR and PLR, along with low LMR, before treatment can help reduce the risk of progression or recurrence following ICI therapy. We recommend further high-quality, large-scale studies to confirm the effectiveness of tracking changes in NLR, PLR, and LMR in evaluating the immune response to tumors. Moreover, we propose utilizing a combination of other tumor markers to customize treatment strategies, improving the outlook and survival rates of patients with GI malignancies and enabling personalized tumor therapy.

## Data availability statement

All data included in this study are available upon request by contact with the corresponding author.

## Author contributions

ST: Data curation, Methodology, Writing – original draft, Writing – review & editing. QZ: Data curation, Methodology, Writing – review & editing. WZ: Data curation, Writing – review & editing. CX: Data curation, Writing – review & editing. MZ: Methodology, Writing – review & editing. WF: Funding acquisition, Supervision, Writing – review & editing.
